# National population mapping from sparse survey data: A hierarchical Bayesian modeling framework to account for uncertainty

**DOI:** 10.1073/pnas.1913050117

**Published:** 2020-09-14

**Authors:** Douglas R. Leasure, Warren C. Jochem, Eric M. Weber, Vincent Seaman, Andrew J. Tatem

**Affiliations:** ^a^WorldPop, Geography and Environmental Science, University of Southampton, Southampton SO17 1BJ, United Kingdom;; ^b^National Security Emerging Technologies Division, Oak Ridge National Laboratory, Oak Ridge, TN 37830;; ^c^Global Development Division, The Bill and Melinda Gates Foundation, Seattle, WA 98109

**Keywords:** demography, international development, Bayesian statistics, remote sensing, geographic information systems

## Abstract

Population estimates are critical for government services, development projects, and public health campaigns. Such data are typically obtained through a national population and housing census. However, population estimates can quickly become inaccurate in localized areas, particularly where migration or displacement has occurred. Some conflict-affected and resource-poor countries have not conducted a census in over 10 y. We developed a hierarchical Bayesian model to estimate population numbers in small areas based on enumeration data from sample areas and nationwide information about administrative boundaries, building locations, settlement types, and other factors related to population density. We demonstrated this model by estimating population sizes in every 10- m grid cell in Nigeria with national coverage. These gridded population estimates and areal population totals derived from them are accompanied by estimates of uncertainty based on Bayesian posterior probabilities. The model had an overall error rate of 67 people per hectare (mean of absolute residuals) or 43% (using scaled residuals) for predictions in out-of-sample survey areas (approximately 3 ha each), with increased precision expected for aggregated population totals in larger areas. This statistical approach represents a significant step toward estimating populations at high resolution with national coverage in the absence of a complete and recent census, while also providing reliable estimates of uncertainty to support informed decision making.

## Significance

High-resolution population estimates are essential for government planning, development projects, and public health campaigns, but countries where this information is most needed are often where recent national census data are least available. We present a modeling framework that combines recent neighborhood-scale microcensus surveys with national-scale data from satellite images and digital maps to estimate population sizes for every 100-m grid square nationally. We present a case study from Nigeria where population estimates with national coverage were produced using household survey data from 1,141 locations. This work represents a significant step toward achieving high-resolution population estimates with national coverage from sparse population data while providing reliable estimates of uncertainty at any spatial scale.

Accurate population estimates are critical for delivering government services, planning development projects, and implementing public health campaigns. These data are typically obtained through a national population and housing census. However, it is in resource-poor and conflict-affected countries, where such datasets are most needed, that recent censuses have not been able to be conducted. The Democratic Republic of Congo has not conducted a census since 1984, yet effective interventions for recent Ebola outbreaks needed accurate estimates of vulnerable population sizes in affected areas. Polio eradication efforts and yellow fever vaccination campaigns in Nigeria are based on census results from 2006. Even where censuses are conducted at standard 10-y intervals, these population estimates can quickly become inaccurate at local scales. In each of these examples, vulnerable populations could be better served if accurate up-to-date population estimates were available at high spatial resolution for specific age and sex groups along with reliable measures of uncertainty to support effective decision making and planning.

There are a variety of approaches for estimating current population sizes when census results are outdated, incomplete, or inaccurate. The United Nations Population Division produces annual updates from projection models that incorporate fertility, mortality, and migration information (1, 2), but these national-level estimates do not account for subnational population patterns. Satellite imagery and other geospatial data have been used to disaggregate population totals from administrative units to create gridded population estimates at a higher resolution, e.g., 100-m grid cells (3), but in many countries this still relies on projections of outdated census results. Alternative approaches are now available in which population counts are collected in small randomly selected areas and these spatially limited but recent survey data (which we call a “microcensus”) are used in combination with geospatial datasets to map predicted population sizes nationally (4). This approach was recently applied to support polio eradication efforts in northern Nigeria where settlement maps derived from satellite imagery were used to extrapolate microcensus results (5).

With all of these approaches, it remains a challenge to accurately account for uncertainty (i.e., patterns of population density that are not well explained by a given population model). It is important that uncertainty intervals accurately reflect uncertainty in the population estimates that may arise from projections, spatial variations in population density, relatively small sample sizes inherent in microcensus data, and other sources. For example, a vaccination campaign may want to plan resources to achieve 90% certainty that the target population will be covered. In this case, the accuracy of the uncertainty intervals may be more important than the mean population estimate. A modeling framework is needed that can utilize appropriate data when they are available to map populations while providing robust estimates of uncertainty.

Advances in Bayesian statistics (6) provide the building blocks necessary to customize models for specific microcensus or other population data. Specifically, hierarchical population models that are commonly used in ecology (7, 8) provide a methodological foundation for mapping populations in data-poor environments and accounting for uncertainty (9, 10). These models can include geospatial covariates as predictors of population density and can easily be extended to accommodate complex relationships such as non-Gaussian error structures, random effects, age structure, observer error, spatial and temporal autocorrelation, and nonlinear models. Each of these topics have important benefits for population modeling and decision making.

**Fig. 1.**
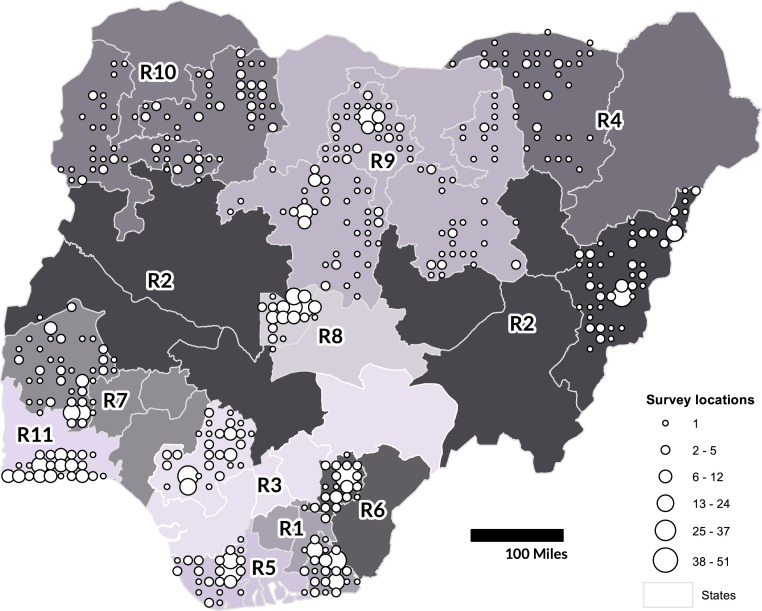
Map of Nigeria showing locations of microcensus surveys as the number of survey locations within each 20-km grid cell. Labeling (R1 to R11) and shading of states indicate regions used for modeling.

Our objective was to construct a Bayesian framework for population mapping using limited microcensus survey data to 1) produce gridded population estimates at 100-m resolution nationally using geospatial covariates and 2) quantify the uncertainty for these estimates and aggregated population totals (e.g., state population totals). We demonstrate the approach here using spatially limited survey data from Nigeria (Fig. 1) to map the population nationally.

## Results

We developed a Bayesian modeling framework that combines population data from recently conducted microcensus surveys with several geospatial covariates. A hierarchical structure was used for this model that enabled it to borrow strength in estimating population densities across settlement types and administrative units. For the Nigeria application, through using geospatial covariates that covered the entire country on a consistent spatial grid, we were able to predict population sizes in all unobserved areas. One of the most important covariates was a settlement map derived from high-resolution satellite imagery that identified settled areas and classified them as nonresidential, rural, or several categories of urban areas (5, 11). Depending on availability of microcensus survey data from a state, population estimates had average error rates from 67 to 92 people per hectare (mean of absolute residuals or 43% based on scaled residuals) for out-of-sample predicted population densities. Full results from the model are available for download (12) including a raster of gridded estimates and a Structured Query Language database containing 10,000 Markov chain Monte Carlo (MCMC) samples from the predicted posterior distribution for each grid cell.

### Population Density.

**Fig. 2.**
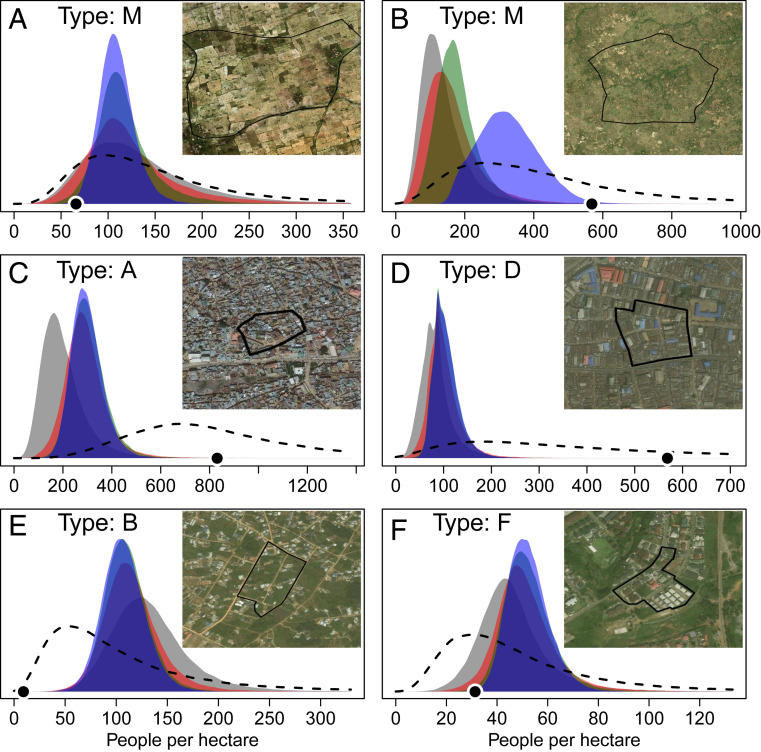
(*A–F*) Posterior probability distribution for random intercepts $\alpha_{t,r,s,l}$ and estimates of population density $D_{i}$ (dashed line) for six microcensus clusters. Gray represents densities for a settlement type nationally (Eq. **7**). Red represents densities for a settlement type within a region (Eq. **6**). Green represents densities for a settlement type within a state (Eq. **5**). Blue represents densities for a settlement type within a local government area (Eq. **4**). Dots represent observed population densities from microcensus surveys. Settlement types shown include rural (M) and several urban types (A, B, D, and F).

Estimates of population density in each microcensus cluster (Fig. 2) were based on a hierarchical random intercept using settlement types and administrative units (Eqs. **4**–**7**) plus the additive effects of geospatial covariates (Eq. **3**). These estimates may be similar to the mean population density for a settlement type nationally (Fig. 2*A*), but in many cases population densities in specific areas differ from the mean (Fig. 2 *B*–*F*). For example, in some rural microcensus clusters in Ebonyi state, population densities were much higher than expected (possibly due to housing for agricultural workers) and there was a high degree of residual variation in space (Fig. 2*B*). Urban areas (type A) of Kano state had higher population densities than expected for the settlement type nationally, and in the cluster shown in Fig. 2*C*, covariates indicated that the population density was even higher than expected (i.e., the dashed distribution is greater than the blue intercept distribution). In urban areas (type D) of Lagos state, there was extremely high variation in population densities that was not well explained by covariates in the model, but $\sigma_{t,r,s,l}$ captured this residual variation well (Fig. 2*D*). Some urban areas (type B) in Akwa Ibom state had slightly lower population densities than the national average for the settlement type. The microcensus cluster shown in Fig 2*E* had covariate values suggesting an even lower density and the observed density was at the low end of this posterior distribution. The federal capital territory of Abuja had slightly higher than average densities for peri-urban areas (type F), but the covariates in the microcensus cluster shown in Fig. 2*F* suggested lower than average densities which matched well with the observation in that cluster.

### Population Totals.

The estimates of population density were made on a 100-m spatial resolution grid. The total population in each grid cell was estimated as a Poisson process (described in *Materials and Methods*) of the density and settled area. By aggregating these grid cells and their posterior distributions we can derive posterior distributions for population totals in administrative units (Table 1) or custom-drawn polygons. By aggregating all grid cells, the total population of Nigeria was estimated to be 179,876,056 (95% CI: 160,361,328 to 207,626,890).

**Table 1.** Example state-level population totals

State	Population	Lower	Upper
Abuja	3,838,085	3,311,346	4,457,200
Borno	5,599,020	2,687,960	10,993,263
Kano	13,704,940	11,836,661	16,085,987
Kaduna	8,623,416	7,420,205	10,138,022
Lagos	9,381,532	7,221,440	13,144,086
Ogun	9,417,916	6,285,736	14,275,008
Sokoto	5,186,534	3,325,988	7,911,709

Population estimates are mean and 95% credible intervals from derived posterior distributions. No microcensus data were available from Borno, Ogun, or Sokoto states.

### Covariate Effects.

Three geospatial covariates included in the model (Eq. **3**) had significant positive effects at the 95% confidence level and three covariates did not (Table 2). Gridded population estimates from WorldPop ($x_{1}$) had a positive relationship with log population densities. Significant positive effects were also detected for school densities ($x_{2}$) and household sizes ($x_{3}$). Residential area ($x_{5}$) within a 1-km radius had a detectable negative effect, but only at the 80% confidence level. Total settled area ($x_{4}$) and nonresidential settlement ($x_{6}$) within a 1-km radius did not have significant effects.

**Table 2.** Estimated covariate effects (untransformed $\beta_{k}$) on population densities

	Mean	Lower 95%	Lower 80%	Upper 80%	Upper 95%
$x_{1}$	0.011	0.004	0.007	0.015	0.017
$x_{2}$	0.027	0.013	0.018	0.036	0.041
$x_{3}$	0.147	0.057	0.089	0.206	0.236
$x_{4}$	−0.007	−0.027	−0.020	0.007	0.014
$x_{5}$	−0.011	−0.027	−0.021	−0.0003	0.005
$x_{6}$	−0.006	−0.016	−0.013	0.001	0.005

*x*_1_ is WorldPop Global population estimates; *x*_2_ is school density; *x*_3_ is household size; *x*_4_ is settled area within 1 km; *x*_5_ is residential area within 1 km; and *x*_6_ is nonresidential area within 1 km.

### Model Diagnostics.

**Fig. 3.**
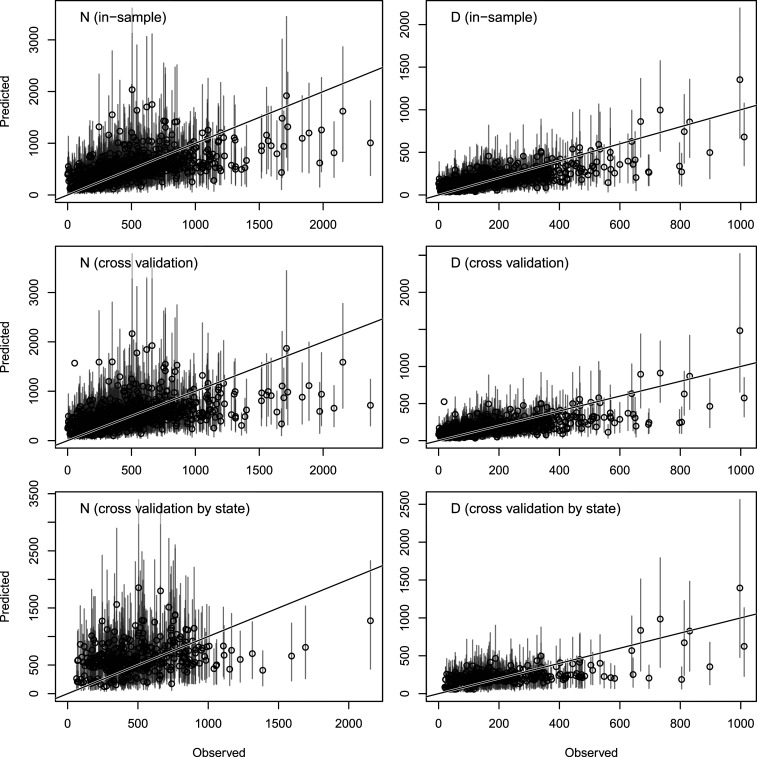
Observed population totals (*N*) and population densities (*D*) in surveyed microcensus clusters versus model predictions. *Top* row shows predictions from the full model, *Middle* row shows random cross-validation, and *Bottom* row shows state-by-state cross-validation results. Diagonal lines are 1:1 lines where predictions equal observations.

Model diagnostics were based on predicted values for each microcensus cluster (i.e., average area = 3 ha). Graphically comparing observed populations to predictions (Fig. 3) showed that cross-validation predictions were similar to in-sample predictions, suggesting that the model performed fairly well in areas without survey data. Estimates of total population size tended to be less accurate than estimates of population density ($r^{2}$ of 0.46 vs. 0.26). This may be due to inaccurate or outdated estimates of settled area from the settlement map. The 95% credible intervals for out-of-sample predictions included the observed values 94.5% of the time, indicating that the model error structure was robust. A summary of observed populations in microcensus clusters compared to posterior predictions is provided in *SI Appendix*, Table S2.

Analysis of residuals (Table 3) indicated a slight positive bias driven by the large number of clusters with low populations where the model tended to overestimate slightly. An assessment of model fit by settlement type is provided in *SI Appendix*, Table S1. Imprecision was relatively high as expected for population predictions in such small spatial areas (i.e., microcensus survey clusters with about 3 ha of settlement). Imprecisions are expected to decrease when gridded population estimates are aggregated to derive population totals for larger areas (e.g., wards, local government areas, or states). Residuals did not indicate strong spatial autocorrelation, suggesting that the random intercept and spatial covariates adequately accounted for spatial effects (*SI Appendix*, Fig. S1). Cross-validated model predictions in states where data were withheld from the model had the highest inaccuracy but average error was only 92 people per hectare (43%) with an imprecision of 121 people per hectare (54%) at a spatial scale of about 3 ha, so predictions were still informative.

**Table 3.** Analysis of residuals for in-sample posterior predictions and out-of-sample cross-validations (X-val)

Parameter	Prediction	Bias	Imprecision	Inaccuracy	$r^{2}$
$N_{i}$	In sample	34 (0.06)	252 (0.50)	179 (0.38)	0.38
$N_{i}$	X-val random	36 (0.04)	284 (0.57)	199 (0.43)	0.26
$N_{i}$	X-val state	121 (0.11)	313 (0.54)	257 (0.43)	0.08
$D_{i}$	In-sample	7 (0.06)	86 (0.50)	61 (0.38)	0.57
$D_{i}$	X-val random	8 (0.04)	96 (0.57)	67 (0.43)	0.46
$D_{i}$	X-val state	24 (0.11)	121 (0.54)	92 (0.43)	0.40

Residuals (predicted minus observed) were calculated based on the mean of the posterior predicted distribution. Bias is the mean of residuals; imprecision is the SD of residuals; inaccuracy is the mean of absolute residuals; *r*_2_ is the squared Pearson correlation coefficient for observed versus predicted values. Values in parentheses are based on scaled residuals (residual/predicted).

MCMC chains for all parameters in the full model reached convergence (see MCMC trace plots, available in Figshare at http://dx.doi.org/10.6084/m9.figshare.12902492). Population estimates for a few clusters did not fully converge in three cross-validation runs due to long right tails. We accepted the risk of slightly conservative estimates of cross-validated model fit.

## Discussion

Accurate, spatially detailed, and up-to-date population data are a key component for planning and monitoring public health and development projects, among many other uses. A number of high-resolution gridded population datasets exist (13–17), but these approaches also depend on up-to-date census data or accurate projections. In contrast, here we demonstrate a modeling framework that produces high-resolution population estimates independent of a census and in the situation where limited recent enumeration has taken place. The approach uses a limited set of recent observed population data collected rapidly and at a fraction of the cost of full national enumeration. The hierarchical modeling framework acknowledges that population densities vary across space and in different socioeconomic contexts. These patterns are represented by data on settlement types, household sizes, and other factors. The model is estimated using Bayesian techniques which enables confidence in population estimates to be quantified based on posterior probability distributions. The predictions and associated uncertainty measures are produced for every 100-m grid square in the country. This fine resolution gives users great flexibility in estimating populations for any defined region of interest or level of aggregation. The uniform grid also allows the population data to be integrated with other datasets, such as local estimates of age–sex structure.

The uncertainty estimates of the predicted population are both an advantage and a challenge of our approach. It is important to consider the uncertainty in the population estimates to use them most effectively. Census data are known to have inaccuracies and to quickly become outdated in some situations, but these uncertainties are rarely quantified or acknowledged, so data users may not be accustomed to considering uncertainty. We suggest that the uncertainty might be used to guide high/low scenarios in planning. For example, the upper estimate could be used when allocating vaccine to local distribution centers during a national vaccine campaign to minimize the chances of vaccine shortages. Not only does population vary across space, the uncertainty does as well, and these patterns can help guide future data collection to improve the model.

We are not implying that population estimates are extremely precise at the 100-m scale (they are not). We are advocating an approach that preserves the ability of end users to aggregate 100-m grid cells to produce population estimates for any geographic area and that provides robust probabilistic estimates of uncertainty at any spatial scale. We have provided tools that allow end users to interact with these Bayesian posterior predictions at the 100-m grid level or aggregated to any larger area while maintaining proper estimates of uncertainty (12, 18).

### Limitations and Model Extensions.

The model assumed that no people lived in areas classified as nonresidential settlements from the LandScanHD settlement map (11). This was necessary because no microcensus surveys were conducted in these areas and borrowing information from residential settlement types, where population densities were potentially much higher, would produce biased estimates of population densities in commercial and industrial areas. Ongoing microcensus surveys have targeted nonresidential areas and have found some populations associated with industrial worker housing and slum areas, and we acknowledge that the current model excludes populations in these areas.

This model also assumed that population sizes were observed without error during microcensus surveys. Observation error will probably result in underestimation of population sizes (i.e., omissions of people are more likely than duplicates) and this effect likely varies among settlement types (e.g., undercounts may be more likely in informal settlements and slums). The assumption of perfect observations could be relaxed if repeat surveys were conducted in some microcensus clusters. There is robust literature from ecology to correct for this bias and to account for the resulting uncertainty in overall population estimates (7). Working within a Bayesian framework gives the flexibility to incorporate an observation submodel when appropriate data become available.

The maps of settled areas and settlement types are critical pieces of information for this model, but the imagery used for settlement mapping was from 2014 and sometimes earlier. This likely resulted in underestimation of populations in areas where urban expansion has occurred. A measurement error submodel (6) could be used to account for this discrepancy using covariates like change in nighttime lights across the time period between settlement mapping and microcensus surveys. Again, the Bayesian framework provides the necessary flexibility to develop this submodel.

Looking to the future, as additional microcensus data continue to be collected, it will be necessary to extend this modeling framework to be a time series model. This will account for temporal autocorrelation in microcensus data allowing multiple years of sparse data to be incorporated into a single model. This will avoid the need for collecting an entirely new microcensus dataset and fitting a new independent model. Instead, information from old microcensus data can be incorporated with newer data. This approach has potential to support a “living census” in which ongoing microcensus data are combined with existing national household survey data to estimate populations at high resolution for improved planning for government services and development projects and to support preparations for a national census. There is a large body of work on Bayesian time series models that could be applied for this purpose (6–8).

### Conclusions.

Population data are key for governments and nongovernmental organizations to plan and evaluate development projects. Beyond operational goals, accurate data on the size, distribution, and demographics of a population are important for understanding the impacts of events such as conflicts, disasters, or improvements in health care, as well as for planning for potential future population trajectories. There is no substitute for the wealth of information beyond simple population counts that can be collected during a national population census, but in the absence of a census or recent extensive enumeration, advances in statistical modeling and geospatial data mean that modeled estimates or population totals, as we demonstrate for Nigeria, can contribute accurate information for providing these vital data.

## Materials and Methods

The analyses undertaken were approved by the ethics and research governance panel of the University of Southampton (submission no. 45895) with oral consent obtained from survey participants.

### Microcensus Data.

Surveys were conducted in 15 of 37 Nigerian states in 2016 and 2017 by eHealth Africa (Fig. 1). We used the total population counts from 1,141 microcensus clusters as input data for our model (Dataset S1). Microcensus survey locations were random samples within each state stratified by settlement type. Each microcensus cluster included about 3 ha of a single settlement type. Settlement types included nonresidential, rural, and four urban categories (Fig. 2).

### Geospatial Covariates.

Administrative boundaries used in the model were local government areas (l), states (s), and regions (r). State and local government area boundaries were obtained from eHealth Africa in September 2018. Regions were groups of states that were thought to share similar population characteristics. Each region contained at least one state with microcensus data. Selecting state groupings is inherently subjective and should be done in collaboration with local experts, stakeholders, and end users. Administrative units are nested hierarchically (i.e., each state is within a single region and each local government area is within a single state).

Settled areas and settlement types were defined in previous research using feature extraction from high-resolution satellite imagery (5, 11). Imagery included WorldView 2 and Pléiades 1A and 1B imagery pan-sharpened at 0.5 m spatial resolution. In areas of overlapping imagery, the best imagery was selected based on date and cloud cover. For about 90% of the project area, imagery dates were 2013 or 2014. Remaining areas required imagery from 2010 to 2012 because of cloud cover. From these data we derived five covariates: settled area (A) in each cluster, settlement type (t), settled area within a 1-km radius ($x_{4}$), residential area in a 1-km radius ($x_{5}$), and nonresidential settled area within a 1-km radius ($x_{6}$). Covariates $x_{4}$, $x_{5}$, and $x_{6}$ were included to provide geographic context of settlements in the area surrounding a survey cluster or prediction location. Covariate $x_{4}$ was scaled based on its mean and SD nationally, whereas covariates $x_{5}$ and $x_{6}$ were scaled based on their mean and SD within a 50-km radius. We scaled $x_{5}$ and $x_{6}$ in this way because we suspected that neighborhood types may not be directly comparable across regions (especially northern versus southern Nigeria). This scaling also reduced correlation with $x_{4}$. Covariates were selected based on assessments of model fit compared to in-sample and out-of-sample observations, but it is beyond the scope of this paper to present formal model comparisons.

Gridded population estimates from WorldPop Global (19) were used to incorporate high-resolution information from the last census in Nigeria. The WorldPop Global project used the “top–down” approach (4) to disaggregate projected census results using random forest models and a large suite of geospatial covariates (3). The resulting gridded population estimates were mapped at 100 m resolution. We scaled the WorldPop Global estimates ($x_{1}$) based on their mean and SD nationally. We averaged these values among pixels within each microcensus cluster. We treated this covariate as an indicator of relative population densities based on geospatial covariates that were in the random forest model. We did not treat the values as population counts, in which case a log transformation may have been appropriate to match the scale of our population density model (Eq. **2**). In the current model, log transformation of this covariate did not have a significant impact on overall model fit.

Schools in Nigeria were mapped by eHealth Africa from 2017 to 2018. We calculated school densities ($x_{2}$) within a 1-km radius of each 100-m grid cell. Covariate $x_{2}$ was scaled using its mean and SD within a 50-km radius. We scaled this covariate within a 50-km moving window because what constitutes a “high density” of schools varies by region and this distinction was lost when the covariate was scaled nationally. This also helped to control for possible differences in school mapping effort in different regions.

We mapped household sizes ($x_{3}$) at 100 m resolution nationally by interpolating Demographic Health Survey results from 2013 (20) to fill gaps between survey areas. We scaled these numbers based on their mean and SD nationally. One key reason for including this covariate was to account for a strong north–south gradient in household sizes, with significantly more people per household in northern Nigeria than in southern Nigeria.

### Hierarchical Bayesian Model.

At their most basic, microcensus surveys produce counts of people $N_{i}$ in each survey cluster i. This can be modeled with the Poisson process model

$$N_{i}∼Poisson\left(D_{i}A_{i}\right),$$ [1]

where $D_{i}$ is population density in the cluster and $A_{i}$ is the total settled area in the cluster. We modeled population density as

$$D_{i}∼LogNormal\left({\bar{D}}_{i},\sigma_{t,r,s,l}\right),$$ [2]

where ${\bar{D}}_{i}$ is the population density (log-scale) expected based on geospatial covariates at location i, and $\sigma_{t,r,s,l}$ quantifies random variations in population densities that were not explained by the covariates. The indexing by t, r, s, and l is explained below. The log-normal in Eq. **2** provides overdispersion for the Poisson distribution in Eq. **1** to adequately capture residual variation in observed counts. Our choice of log-normal is consistent with a posthoc simulation approach previously used in Nigeria to quantify uncertainty (5).

Expected population densities were estimated using a log-linear regression with a random intercept and K geospatial covariates $x_{k}$,

$${\bar{D}}_{i}=\alpha_{t,r,s,l}+\overset{K}{\underset{k=1}{\sum }}\beta_{k}x_{k,i},$$ [3]

where $\alpha_{t,r,s,l}$ is the mean population density for a local government area and $\beta_{k}$ are the effects of geospatial covariates $x_{k,i}$ on population densities in specific locations i. This model assumes that covariates have a linear relationship with $log\left(\bar{D}\right)$.

The random intercept $\alpha_{t,r,s,l}$ was modeled hierarchically by settlement type t, region r, state s, and local government area l:

$$\alpha_{t,r,s,l}∼Normal\left(\mu_{t,r,s},\theta_{t,r,s}\right)$$ [4]

$$\begin{array}{cc} \mu_{t,r,s} & ∼Normal\left(\mu_{t,r},\theta_{t,r}\right)\\ \theta_{t,r,s} & ∼Uniform\left(0,\theta_{t,r}\right) \end{array}$$ [5]

$$\begin{array}{cc} \mu_{t,r} & ∼Normal\left(\mu_{t},\theta_{t}\right)\\ \theta_{t,r} & ∼Uniform\left(0,\theta_{t}\right) \end{array}$$ [6]

$$\begin{array}{cc} \mu_{t} & ∼Normal\left(\mu ,\theta \right)\\ \theta_{t} & ∼Uniform\left(0,\theta \right). \end{array}$$ [7]

The average population density $\alpha_{t,r,s,l}$ for settlement type t in local government area l is drawn from the distribution of average population densities for that settlement type throughout the state (Eq. **4**). This distribution is defined by the statewide mean $\mu_{t,r,s}$ and SD $\theta_{t,r,s}$ from Eq. **5**. The state-level SD $\theta_{t,r,s}$ cannot exceed the regional-level SD $\theta_{t,r}$. The average density for a settlement type statewide $\mu_{t,r,s}$ is drawn from the distribution of average densities for the settlement type in the region; regional densities ($\mu_{t,r}$) are drawn from distribution of densities for the settlement type nationally; and average densities for each settlement type ($\mu_{t}$) are drawn from the distribution of densities among all microcensus clusters.

This hierarchical structure shares information among local government areas and states. For example, if no microcensus data exist for a local government area, the average densities for each settlement type will be estimated based on other local government areas in the state where data were collected. If no microcensus data exist for a state, the estimate will be dominated by data from other states in the region. All regions had at least one state with data.

The hierarchical random intercept also accounts for spatial autocorrelation inherent in data from nearby clusters (*SI Appendix*, Fig. S1). This is particularly important for microcensus data that are often geographically clustered as a result of incomplete state-by-state data collection. Geostatistical models are often used for this purpose but they are much more computationally demanding and often less flexible as a result.

Residual variation $\sigma_{t,r,s,l}$ was estimated for every local government area using a hierarchical structure similar to Eqs. **4**–**7**, except it used half-normal distributions (i.e., truncated to be greater than zero):

$$\sigma_{t,r,s,l}∼\text{Half-Normal}\left(\eta_{t,r,s},\epsilon_{t,r,s}\right)$$ [8]

$$\begin{array}{cc} \eta_{t,r,s} & ∼\text{Half-Normal}\left(\eta_{t,r},\epsilon_{t,r}\right)\\ \epsilon_{t,r,s} & ∼Uniform\left(0,\epsilon_{t,r}\right) \end{array}$$ [9]

$$\begin{array}{cc} \eta_{t,r} & ∼\text{Half-Normal}\left(\eta_{t},\epsilon_{t}\right)\\ \epsilon_{t,r} & ∼Uniform\left(0,\epsilon_{t}\right) \end{array}$$ [10]

$$\begin{array}{cc} \eta_{t} & ∼\text{Normal}\left(\eta ,\epsilon \right)\\ \epsilon_{t} & ∼Uniform\left(0,\epsilon \right). \end{array}$$ [11]

Minimally informative priors were defined as

$$\begin{array}{c} \beta_{k}∼Normal\left(0,5\right)\\ \mu ∼Normal\left(0,31.6\right)\\ \eta ∼\text{Half-Normal}\left(0,31.6\right)\\ \theta ∼Uniform\left(0,1,000\right)\\ \epsilon ∼Uniform\left(0,1,000\right). \end{array}$$ [12]

The model was estimated with MCMC methods in JAGS (Just Another Gibbs Sampler) (21) using the R package runjags (22). The model code is available in Dataset S2. Convergence of MCMC chains was assessed using the Gelman–Rubin statistic and values less than 1.1 were interpreted as indicating convergence (6). Trace plots of MCMC chains are provided in Figshare (http://dx.doi.org/10.6084/m9.figshare.12902492). Spatial autocorrelation in model residuals was assessed using semivariograms and Moran’s I statistics (*SI Appendix*, Fig. S1). Model fit was assessed using 10-fold cross-validation where the model was refitted 10 times, each time withholding a random 10% of survey clusters until all had been held out once. Model fit for predictions into unsampled states was assessed by holding out data from an entire state (where at least two states from a region had samples), refitting the model each time. For predicted population sizes and densities, we evaluated bias (mean of residuals), imprecision (SD of residuals), inaccuracy (mean of absolute residuals), and r-squared values (squared Pearson correlation coefficient). Uncertainty in model predictions accounted for uncertainty at all levels of the model: parameter uncertainty, Poisson process error for population counts, and log-normal process error for population densities. Uncertainties in model predictions did not account for error introduced by processes that were not explicitly modeled such as observer error, settlement mapping, and movements of people.

## Supplementary Material

Supplementary File

Supplementary File

Supplementary File

## Data Availability

All data discussed in this paper are available to readers. Input data and model code are provided in Datasets S1 and S2, and MCMC trace plots are available in Figshare (http://dx.doi.org/10.6084/m9.figshare.12902492). Full model results are available for download (12) and can be explored on an interactive web application (18). Model outputs (.csv, .tif, .sql, XYZ web map tiles) data have been deposited in the WorldPop Open Population Repository (http://dx.doi.org/10.5258/SOTON/WP00655). All study data are included in this paper and *SI Appendix*.
