# Supplementary material for: National population mapping from sparse survey data: A hierarchical Bayesian modeling framework to account for uncertainty
Source: Proc Natl Acad Sci U S A. 2020 Sep 14;117(39):24173–9. doi: 10.1073/pnas.1913050117 (PMC7533662; doi:10.1073/pnas.1913050117)
Supplement: Supplementary File [file pnas.1913050117.sapp.pdf]

PNAS
www.pnas.org

Supplementary Information for

National population mapping from sparse survey data: a hierarchical Bayesian modelling framework to account for uncertainty

Douglas R. Leasure, Warren C. Jochem, Eric M. Weber, Vincent Seaman, Andrew J. Tatem

Douglas R. Leasure
Email: D.R.Leasure@soton.ac.uk

This PDF file includes:

Supplementary text
Figure S1
Table S1
Table S2
Legends for Datasets S1 to S3
SI References

Other supplementary materials for this manuscript include the following:

Datasets S1 to S3

www.pnas.org/cgi/doi/10.1073/pnas.1913050117

Supplementary Information Text

We have included detailed model results to give readers additional context to interpret the model results and the inputs needed to independently replicate the model.

Dataset S1 provides the input data to the JAGS model and the Dataset S2 provides source code for the model (JAGS language). These datasets can be used to independently run the model for further assessments. They could also be used as a starting point for modifying the model to apply it in new contexts.

The MCMC traceplots (Dataset S3) show the marginal posterior distributions for all parameters in the model. The traceplots do not show indications of influential priors (e.g. truncated tails of posteriors) or identifiability issues (e.g. parallel unconverged MCMC chains for a parameter) that could have arisen from interactions between the hierarchical intercepts and the hierarchical variances.

We included a breakdown of model assessments by settlement type in Table S1. It is clear from these results that the model performs differently in different settlement types, but even where the model is imprecise, the 95% prediction intervals include the observed values about 95% of the time. This indicates that the error structure is robust and it emphasizes the importance of accounting for uncertainty in population estimates because the mean predictions are often imprecise at small spatial scales, where population densities vary significantly across space, and in data-poor regions.

Table S2 shows how the observed population compared to the Bayesian posterior prediction at each location. These results suggest that the model's variance structure is adequately accounting for uncertainty in population estimates because the expected proportions of observations fall outside the prediction intervals. For example, about 5% of observations were less than the 5th quantile of the posterior prediction for the location. This trend was evident for in-sample and out-of-sample observations for predicted population densities and counts.

To ensure that our model structure (particularly the hierarchical random intercept) was accounting for spatial autocorrelation, we assessed Moran's I of the model residuals (Figure S1) for ranges up to 100 km. We found no spatial autocorrelation except one significant value at the smallest range size for the raw residuals of total population size, but there were no significant Moran's I values for standardized residuals or when population densities were treated as the response variable (raw or standardized residuals). We interpreted these results as indicating that the model structure was adequately accounting for spatial autocorrelation, leaving little spatial structure in the residuals.

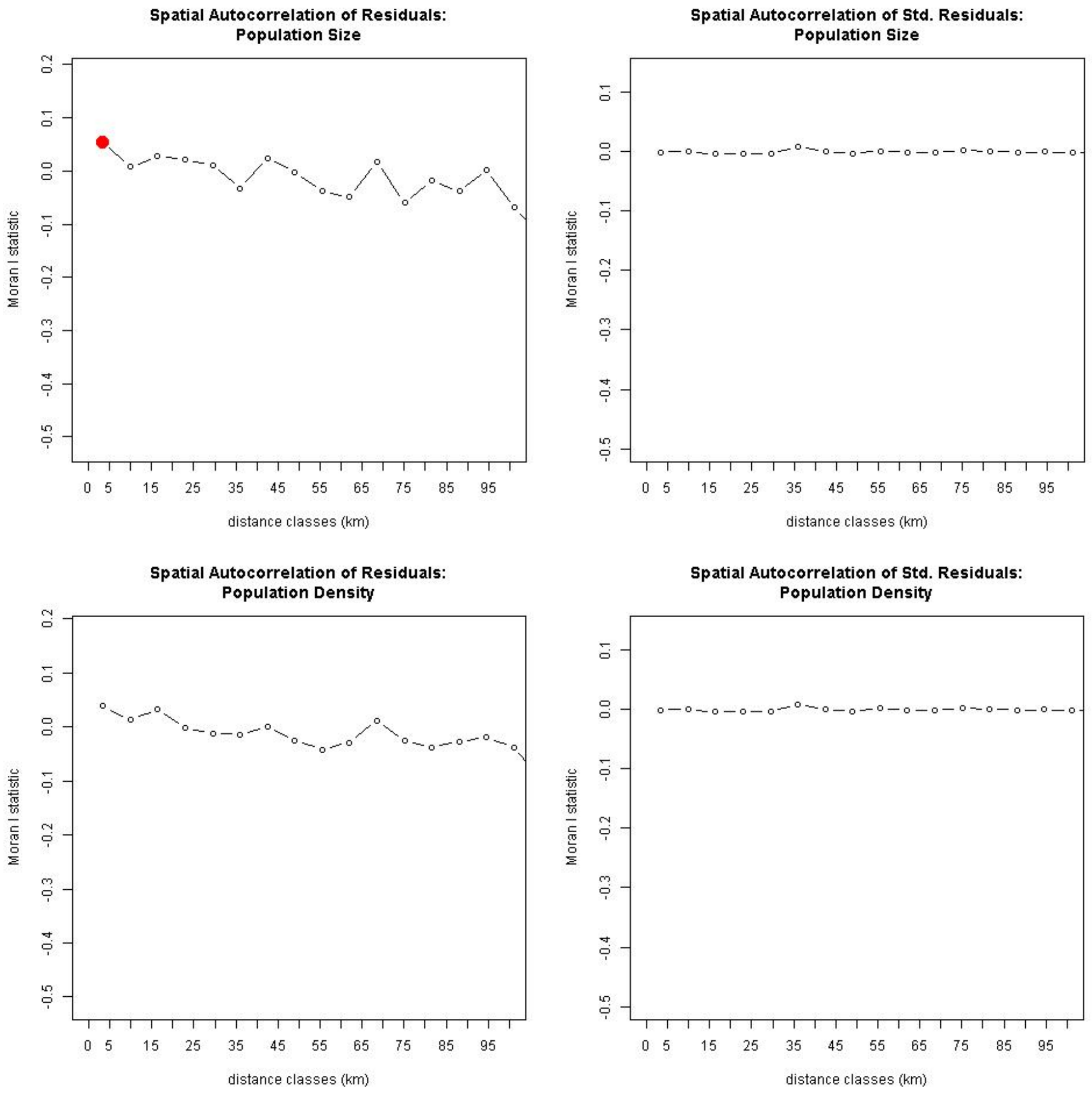

Fig. S1. Spatial correlograms showing Moran's I statistics calculated at ranges from 0 to 100 km to assess spatial autocorrelation of model residuals. Red dots indicate a statistically significant Moran's I statistic ($P<0.05$). Correlograms were constructed from residuals in population size (N) and population density (D) using raw residuals as well as standardized residuals.

Table S1. Model fit assessment for each settlement type separately. “Response” indicates the response variable being assessed (N=population count, D=population density). “Sample” indicates whether the assessment was based on in-sample or out-of-sample observations. “Type” indicates the settlement type. “n” indicates the sample size. “Obs.mean” is the mean of observed data and “Obs.sd” is the standard deviation. “Obs.inCI” is the proportion of the observations that fell within the 95% prediction intervals. “Bias” is the mean of the residuals. “Impr” is imprecision calculated as the standard deviation of residuals. “Inac” is inaccuracy calculated as the mean of the absolute residuals. “r2” is the r-squared value calculated as the squared Pearson correlation coefficient. Residuals were calculated as the mean of the posterior prediction minus the observed value.

Response	Sample	Type	n	Obs.mean	Obs.sd	Obs.inCI	Bias	Impr	Inac	r2
N	in-sample	A	117	677	412.5	0.966	22.8	314.6	216	0.419
N	in-sample	B	164	454.9	245.8	0.951	57.6	266.8	187.7	0.149
N	in-sample	D	106	311	261.4	0.962	70.4	222.4	165.6	0.285
N	in-sample	F	113	171	129.7	0.982	12.3	102	79.3	0.408
N	in-sample	M	641	470.5	300.9	0.964	27.5	258.5	189.2	0.286
N	cross-validation	A	117	677	412.5	0.897	14.1	368.3	253.2	0.223
N	cross-validation	B	164	454.9	245.8	0.902	64.1	286.5	200.6	0.113
N	cross-validation	D	106	311	261.4	0.915	79.7	282.4	195	0.061
N	cross-validation	F	113	171	129.7	0.885	13.8	110.2	85.8	0.327
N	cross-validation	M	641	470.5	300.9	0.9	29.1	286.2	209.5	0.173
D	in-sample	A	117	264.7	201.6	0.966	9.6	116	79.3	0.68
D	in-sample	B	164	190.3	145.7	0.951	10.2	104.5	72.7	0.486
D	in-sample	D	106	103.2	90.7	0.962	19.6	71.3	52.5	0.386
D	in-sample	F	113	49.4	32.8	0.982	2.6	30.9	23.8	0.112
D	in-sample	M	641	158.7	104.5	0.964	5	82.6	61.8	0.384
D	cross-validation	A	117	264.7	201.6	0.897	5.7	137.9	93.4	0.565
D	cross-validation	B	164	190.3	145.7	0.902	11.5	110.5	77.1	0.427
D	cross-validation	D	106	103.2	90.7	0.915	22.5	91.8	62.3	0.091
D	cross-validation	F	113	49.4	32.8	0.885	3.1	33.6	25.8	0.018
D	cross-validation	M	641	158.7	104.5	0.899	5.1	91.1	68.5	0.239

Table S2. Proportion of observed data points that were less than the reported quantiles from the Bayesian posterior predictions. “Response” indicates the response variable being assessed (N=population count, D=population density). “Sample” indicates whether the assessment was based on in-sample or out-of-sample observations. The remaining columns show the proportion of microcensus locations where the observed data that were less than the reported quantile of the posterior prediction for the location. For example, the “Q5” column shows the proportion of microcensus locations where the observed data were less than the 5th quantile of the posterior prediction for the location.

Response	Sample	Q5	Q10	Q25	Q50	Q75	Q90	Q95
N	in-sample	0.045	0.077	0.197	0.465	0.782	0.952	0.981
N	cross-validation	0.056	0.096	0.214	0.469	0.755	0.926	0.971
D	in-sample	0.046	0.079	0.204	0.466	0.782	0.951	0.981
D	cross-validation	0.059	0.101	0.216	0.469	0.755	0.925	0.970

Dataset S1 (separate file). Input data for the hierarchical Bayesian population model. “N” is the observed number of people in each survey cluster. “A” is the total settled area in the survey cluster. “type” is the settlement type. “region” is a region identifier. “state” is a state identifier. “local” is an identifier for local government area. “x1” is the scaled average of WorldPop Global population estimates in the cluster. “x2” is scaled school density within a 1km radius. “x3” is the scaled average household size. “x4” is scaled settled area within a 1 km radius. “x5” is scaled residential area with a 1 km radius. “x6” is scaled non-residential area within a 1 km radius.

Dataset S2 (separate file). A text file containing JAGS code for the model.

Dataset S3 (separate file). A zip file containing MCMC traceplots (.jpg) for the fitted model. This dataset is available for download from http://dx.doi.org/10.6084/m9.figshare.12902492.
